# Predicting U.S. Tuberculosis Case Counts through 2020

**DOI:** 10.1371/journal.pone.0065276

**Published:** 2013-06-13

**Authors:** Rachel S. Y e l k Woodruff, Carla A. Winston, Roque Miramontes

**Affiliations:** Centers for Disease Control and Prevention, Atlanta, Georgia, United States of America; McGill University, Canada

## Abstract

In 2010, foreign-born persons accounted for 60% of all tuberculosis (TB) cases in the United States. Understanding which national groups make up the highest proportion of TB cases will assist TB control programs in concentrating limited resources where they can provide the greatest impact on preventing transmission of TB disease. The objective of our study was to predict through 2020 the numbers of U.S. TB cases among U.S.-born, foreign-born and foreign-born persons from selected countries of birth. TB case counts reported through the National Tuberculosis Surveillance System from 2000–2010 were log-transformed, and linear regression was performed to calculate predicted annual case counts and 95% prediction intervals for 2011–2020. Data were analyzed in 2011 before 2011 case counts were known. Decreases were predicted between 2010 observed and 2020 predicted counts for total TB cases (11,182 to 8,117 [95% prediction interval 7,262–9,073]) as well as TB cases among foreign-born persons from Mexico (1,541 to 1,420 [1,066–1,892]), the Philippines (740 to 724 [569–922]), India (578 to 553 [455–672]), Vietnam (532 to 429 [367–502]) and China (364 to 328 [249–433]). TB cases among persons who are U.S.-born and foreign-born were predicted to decline 47% (4,393 to 2,338 [2,113–2,586]) and 6% (6,720 to 6,343 [5,382–7,476]), respectively. Assuming rates of declines observed from 2000–2010 continue until 2020, a widening gap between the numbers of U.S.-born and foreign-born TB cases was predicted. TB case count predictions will help TB control programs identify needs for cultural competency, such as languages and interpreters needed for translating materials or engaging in appropriate community outreach.

## Introduction

The primary responsibility for treating, and oftentimes diagnosing, patients with tuberculosis (TB) and latent tuberculosis infection (LTBI) falls to state and local TB control programs [1]. The number of current and future TB cases is an important measure of burden on TB control programs and has implications for setting priorities among TB prevention and control efforts. Health departments not accustomed to serving large foreign-born populations may experience an increase in TB cases as a result of increasing numbers of foreign-born residents, who may be arriving from countries with high rates of TB. As foreign-born persons make up an increasing proportion of TB cases in the United States, predicting TB trends will help TB control programs identify needs for cultural competency, including foreign language speakers and interpreters needed for translating materials or engaging in appropriate community outreach.

Several studies have predicted future TB trends [2], [3], [4], [5], [6], [7], [8], [9], [10] however we are not aware of any published studies that predict future TB case counts in the United States among foreign-born persons from specific countries of birth. As progress continues in decreasing the burden of TB in the United States [11], understanding TB trends among the foreign-born groups that make up the largest proportion of TB cases will assist TB control programs in concentrating limited resources where they can provide the greatest impact. The purpose of this study was to help guide TB control and program planning. We achieved this objective by predicting the numbers of U.S. TB cases among U.S.-born, foreign-born and foreign-born persons from selected countries of birth.

## Methods

### Study Participants

The National Tuberculosis Surveillance System (NTSS) collects annual TB case information from 60 reporting areas including the 50 states and the District of Columbia [12]. NTSS data consist of demographic, clinical, and risk factor information. Nativity (native or U.S.-born vs. foreign-born) is determined based on a person’s self-reported country of birth and U.S. citizenship status at birth, and follows U.S. Census definitions [13], [14]. A reported case is confirmed as TB according to laboratory or clinical criteria [13], [15].

### Ethics Statement

Data used in this evaluation were collected as part of routine disease surveillance activities, and the project was not considered to be human subjects research requiring Institutional Review Board approval.

### Trend and Prediction Analyses

Annual aggregate NTSS data from January 1, 2000, through December 31, 2010, were analyzed to determine the proportion and number of U.S. TB cases by nativity and to examine current trends and predict future case counts for all TB cases, U.S.-born TB cases, foreign-born TB cases, foreign-born TB cases from the top 5 countries of birth, and foreign-born TB cases from countries of birth other than the top 5.

TB surveillance data from years 2000 through 2010 were selected to reflect recent trends, namely the slower decline of TB rates beginning in 2000 compared to earlier declines [16], and because 2010 was the most recent data available in 2011, the time of analysis. Case counts were log-transformed to better fit the statistical assumptions of linear regression which was performed on the log-transformed counts as the dependent variable with year as the independent variable (regression results were back-transformed into case counts). Regression models calculated predicted annual case counts and 95% prediction intervals from 2011 through 2020. In secondary analyses, we included all available data from 1993–2010 to assess the choice of baseline year. Regression analyses were conducted using SAS (version 9.2; SAS Institute, Cary, NC). The regression line slope was considered significantly different from 0 at p<0.05. The R^2^ coefficient of determination was examined to assess the proportion of variability in case count that may be explained by year.

### Joinpoint Analysis

In order to assess the single linear slope assumption in our regression analysis, trends in TB case counts were examined using joinpoint models (Joinpoint version 3.5.2, Bethesda, MD http://surveillance.cancer.gov/joinpoint/). Significant changes in trend, or joinpoints, were identified by fitting a series of piece-wise regression models to 2000–2010 TB data. The number of allowable joinpoints ranged from 0 (a straight line, equivalent to our linear regression model) to 3. Statistically significant changes in trends were detected using a Monte Carlo permutation method. Annual percent change (APC) in log counts and associated 95% confidence intervals (CI) were calculated for each joinpoint segment to characterize and compare trends. APC was considered significantly different from 0 at p<0.05. Segmented linear regression analyses to predict TB cases and 95% prediction intervals from 2011–2020 were conducted on log-transformed case counts for groups for which a significant joinpoint was indicated. Segmented analysis predictions were based on the second segment of the joinpoint analysis.

## Results

From 2000 to 2010 a total of 153,353 cases of tuberculosis were reported in the United States; 45% (69,189) were among U.S.-born persons and 55% (83,625) were among foreign-born persons. TB cases among U.S.-born persons declined 49% from 2000 to 2010. The number of TB cases among foreign-born persons remained stable from 2000 to 2008 (0.6% decline), with an 11% decline in observed counts from 2008 to 2010.

The top 5 countries of birth among foreign-born persons diagnosed with TB for each year from 2000 to 2010 were Mexico, the Philippines, Vietnam, India and China. The greatest numbers of TB cases among foreign-born persons diagnosed in the United States were consistently among persons from Mexico, followed by the Philippines (24% and 11% of all TB cases among foreign-born persons between 2000 and 2010, respectively).

### TB Prediction Results

Regression analysis predicted a decrease between 2010 observed and 2020 predicted counts for TB cases among all persons in the United States as well as TB cases among persons who were U.S.-born, foreign-born, and foreign-born from Mexico, the Philippines, India, Vietnam and China (Table 1). Among all TB cases diagnosed in the United States, the proportion of cases among persons who were foreign-born was predicted to increase from 60% in 2010 to 78% by 2020. The number of TB cases among U.S.-born persons was predicted to decline 47% from 2010 (4,393 observed cases) to 2020 (2,338 predicted cases) and the number of TB cases among foreign-born persons was predicted to decline 6% (6,720 observed cases to 6,343 predicted cases; Table 1). The 95% prediction intervals for TB cases in 2020 among foreign-born persons, foreign-born persons from the top 5 countries, foreign-born persons from countries other than the top 5 and foreign-born persons from Mexico, the Philippines, India and China included the number of cases observed in 2010. U.S.-born persons and foreign-born persons from Vietnam were the only groups for which the 2010 observed case count was higher than the 2020 prediction bounds.

**Table 1:** 2010 observed and 2020 predicted tuberculosis (TB) case counts and 95% prediction intervals.

	2010 Observed TB Case Count	2020 Predicted TB Case Count (95% Prediction Interval)
Total*	11,182	8,117 (7,262–9,073)
U.S.-born*	4,393	2,338 (2,113–2,586)
Foreign-born*	6,720	6,343 (5,382–7,476)
Foreign-born from top 5 countries*	3,755	3,446 (2,900–4,096)
Mexico*	1,541	1,420 (1,066–1,892)
Philippines	740	724 (569–922)
India	578	553 (455–672)
Vietnam*	532	429 (367–502)
China	364	328 (249–433)
Foreign-born from countries other than top 5	2,965	2,901 (2,432–3,459)

* P<0.05 indicating the slope for 2000–2010 regression line was significantly different from zero.

**Figure 1:**
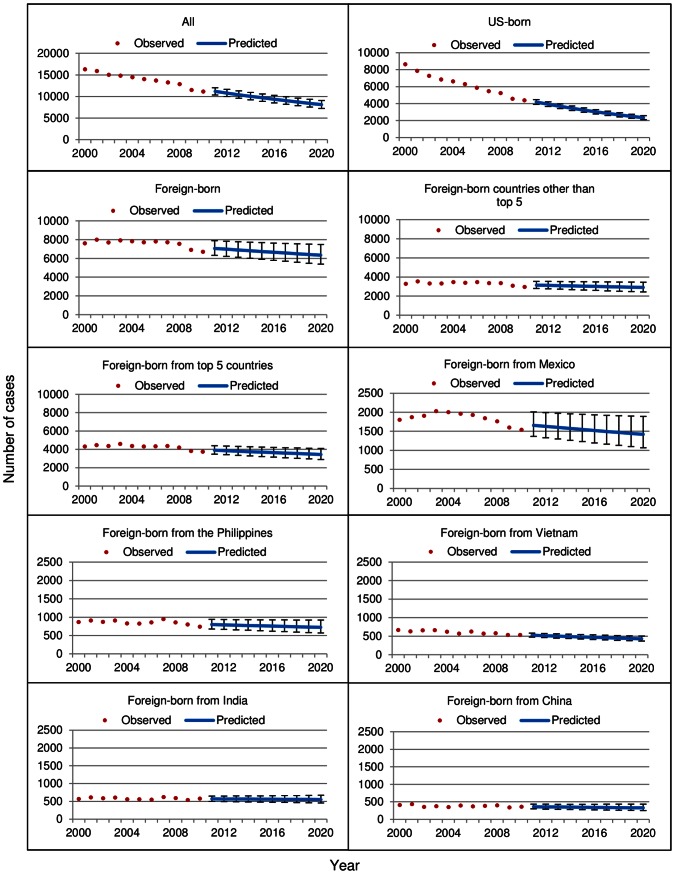
Comparison of observed and predicted tuberculosis (TB) case counts among select groups, United States, 2000–2020. Observed values for 2000–2010 reported to the National Tuberculosis Surveillance System were log transformed and logistic regression was performed on the log transformed counts to generate predicted values for 2011–2020. Vertical bars represent 95% prediction intervals.

Model results indicated a significantly decreasing linear slope by year during 2000–2010 for all sub-groups except foreign-born persons from the Philippines, India, China, and foreign-born persons born in countries other than the top 5. Of the top 5 countries of birth, TB cases among persons born in Mexico were predicted to experience the steepest decline in numbers from 2011 to 2020 (Figure 1). The predicted decline in TB cases among foreign-born persons was influenced by the top 5 countries of birth; the foreign-born slope flattened and statistical significance changed when the top 5 countries of birth were excluded (p = 0.05 compared to p = 0.01 for all foreign born; Figure 1).

Model fit was best for TB case counts from 2000 to 2010 among U.S.-born persons (R^2^ = 0.99), followed by TB cases among all persons (R^2^ = 0.95), foreign-born persons from Vietnam (R^2^ = 0.81), foreign-born persons from the top 5 countries of birth (R^2^ = 0.57), foreign-born persons overall (R^2^ = 0.51), and foreign-born persons from Mexico (R^2^ = 0.41). The model had little predictive value among foreign-born persons from countries of birth other than the top 5 (R^2^ = 0.35) or among foreign-born persons from the Philippines (R^2^ = 0.29), China (R^2^ = 0.29) and India (R^2^ = 0.05), which had flat slopes.

Modeling 1993 through 2010 case counts resulted in predicted increases in case counts by 2020 among foreign-born persons overall (6,720 observed cases in 2010 to 7,123 [95% prediction interval 6,327–8,019] predicted in 2020), foreign-born persons from Mexico (1,541 to 1,719 [95% prediction interval 1,401–2,108]) and foreign-born persons from India (578 to 913 [95% prediction interval 609–1,368]), rather than the decreases predicted when modeling case counts from 2000 through 2010. Results for other countries were qualitatively unchanged.

### Joinpoint Results

**Figure 2:**
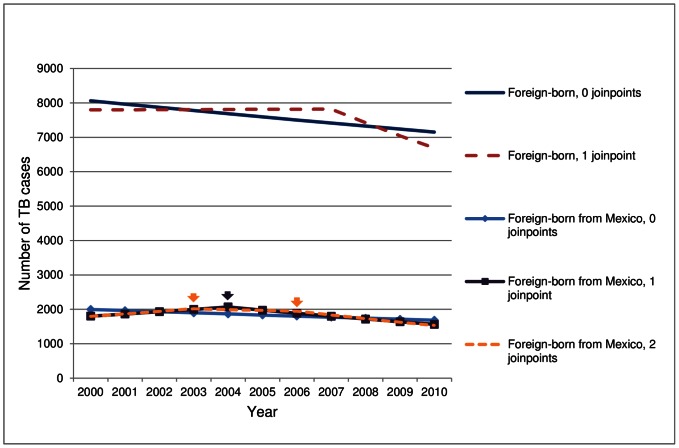
Number of tuberculosis (TB) cases modeled with 0 and 1 significant changes in trend for all foreign-born persons and 0, 1, and 2 significant changes in trend for foreign-born persons from Mexico, 2000–2010. Significant changes in trend were determined using Joinpoint analysis (Joinpoint version 3.5.2, Bethesda, MD http://surveillance.cancer.gov/joinpoint/). Arrows indicate the years in which significant changes in trend for foreign-born persons from Mexico occurred (blue arrow corresponds to model results with a single change in trend and red arrows correspond to model results with 2 changes in trend).

When assessing for possible significant changes in trend, a significant joinpoint was suggested over a single slope model for TB cases among all persons and cases among persons who were foreign-born, foreign-born from countries of birth other than the top 5, foreign-born from the top 5 countries of birth, and foreign-born from Mexico (Table 2). TB cases among all foreign-born persons exhibited similar trends as those who were foreign-born from Mexico when modeled with no joinpoint. However, 1-joinpoint models resulted in a significant decrease in observed trend among all foreign-born persons occurring 3 years later than among foreign-born persons from Mexico (Figure 2, Table 2). Allowing the model to select a maximum of 3 joinpoints resulted in a recommended 2-joinpoint model for Mexico with a significant increase of 3.9% from 2000–2003, a non-significant decrease of 1.2% from 2003–2006, and a significant decrease of 5.6% from 2006–2010. All other recommended models remained the same as shown in Table 2.

**Table 2:** Joinpoint^a^ analysis results for significant change in trend among 2000–2010 TB case counts.

	0 Joinpoint Model^b^	1 Joinpoint Model^b^	
	APC^c^ (95% CI^d^)	Years^e^	APC (95% CI)
Total	−3.5* (−4.1, −2.9)	2000–2008	−2.9* (−3.3, −2.6)
		2008–2010	−7.4* (−10.2, −4.4)
US-born	−6.3* (−6.8, −5.7)		
Foreign-born	−1.2* (−2.1, −0.3)	2000–2007	0.0 (−0.9,1.0)
		2007–2010	−5.1* (−8.2, −1.8)
FB from the top 5 countries	−1.4* (−2.3, −0.5)	2000–2007	−0.2 (−1.3, 1.0)
		2007–2010	−5.3* (−9.4, −1.1)
Mexico	−1.7* (−3.2, −0.2)	2000–2004	3.5* (1.2, 5.8)
		2004–2010	−4.6* (−5.7, −3.4)
Philippines	−1.1 (−2.4, 0.2)		
India	−0.3 (−1.4, 0.7)		
Vietnam	−2.2* (−3.1, −1.4)		
China	−1.0 (−2.4, 0.5)		
FB excluding top 5 countries	−0.9 (−1.9, 0.0)	2000–2008	−0.0 (−1.0, 1.0)
		2008–2010	−7.0* (−15.2, −2.0)

a Joinpoint version 3.5.2, Bethesda, MD http://surveillance.cancer.gov/joinpoint/.

b 0 joinpoint model results are included for all groups analyzed and 1 joinpoint model results are included for those which a significant change in trend was indicated.

c APC = Annual Percent Change in TB case counts.

d CI = Confidence Interval.

e Indicates the year in which a significant change in trend occurred.

* The APC is significantly different from 0 at p<0.05. For the 1 joinpoint model, asterisks indicate which segment(s) had a significant APC. The Philippines, India and China did not have a significant APC result from either 0 or 1 joinpoint models.

Compared to single slope results for 2000–2010, segmented regression resulted in lower predicted TB case counts for 2020 among all persons (5,133 predicted cases [95% prediction interval 4,109–6,411]), foreign-born persons (3,976 predicted cases [3,300–4,791]), foreign-born persons from the top 5 countries (2,147 predicted cases [1,710–2,697]), foreign-born persons from countries other than the top 5 (1,424 predicted cases [982–2,064]) and foreign-born persons from Mexico (982 predicted cases [859–1,122]).

## Discussion

TB cases are predicted to decline among all persons in the U.S. A steeper decline is predicted among U.S.-born than foreign-born persons, which has implications for state and local TB control programs and TB elimination in the U.S. Among the top 5 countries of birth of foreign-born TB patients diagnosed in the United States, our model predicted a significant decrease and 2010 observed case count above the upper bound of the 2020 prediction interval only for foreign-born persons from Vietnam. The remaining countries (Mexico, the Philippines, India, and China) exhibited flatter slopes and 2020 prediction intervals that included the value of the 2010 observed count; consequently our model results for 95% prediction intervals indicate that cases among persons from these countries have the potential to increase or decrease. Modeling case counts from a longer time period (1993 to 2010) resulted in a significant increase in predicted estimates of cases among persons born in India, but ignores the change in the deceleration rate of TB from the 1990s to the 2000s [16]. We primarily presented single slope regression models for comparability across groups. Using a segmented regression approach with more than 1 slope when indicated resulted in even greater predicted declines in TB case counts by 2020. Thus, our predicted estimates from single slope regression models are conservative compared to other types of prediction models.

TB cases among persons who were foreign-born and foreign-born from the top 5 countries of birth experienced significant changes in trend in 2007 resulting in steeper declines during the latter part of the decade. However, among the individual top 5 countries of birth a significant change in TB case count trend from 2000–2010 was indicated only for Mexico. Mexico’s significant changes in trends occurred in 2004 (for a single joinpoint model), or in 2003 and 2006 (for a double joinpoint model). The single joinpoint change in trend among foreign-born from Mexico preceded the significant change in trend among all foreign-born by at least 3 years. Therefore, we hypothesized that the change in cases among persons from Mexico, which accounted for nearly a quarter of all foreign-born TB cases in the United States, influenced the significant change to a decreasing trend among all foreign-born persons. When Mexico was removed from foreign-born, results were consistent with this hypothesis; though the slope of the regression line among foreign-born excluding Mexico decreased in a similar manner as foreign-born including Mexico, the change in trend among foreign-born excluding Mexico occurred in 2008, a year later than it occurred for all foreign-born (data not shown). Thus the decrease in TB cases among foreign-born persons from Mexico influenced the year in which the foreign-born change in trend occurred and, subsequently, will continue to influence TB trends among foreign-born persons in coming years.

In 2001, TB case rates were 5 times higher among foreign-born persons from Mexico than U.S.-born persons, and Mexican-born TB patients were more likely than U.S.-born TB patients to have multidrug-resistant TB [17]. Furthermore, in California and Texas TB morbidity among U.S.-born persons is higher in counties that border Mexico than in the rest of the state [17]. The number of persons from Mexico emigrating to the U.S. declined by 60% from 2006 to 2010 [18], however this decrease has had minimal impact on the percentage of foreign-born TB cases attributed to persons from Mexico [12]. The unique challenges associated with TB among foreign-born persons from Mexico need to be continually addressed if the decline of TB among foreign-born persons is to be accelerated, thereby continuing the decline of TB in the U.S. [17], [19]. Bi-national programs, such as California’s CureTb and Texas’ TB Net, as well as TB control programs for Mexico-born migrant workers and non-immigrants, are necessary to diagnose, treat, and thereby reduce TB cases among foreign-born persons from Mexico.

Immigration, which is directly affected by national policies and socioeconomic factors [20], is an important consideration in the number of TB cases among foreign-born persons [21], [22]. Major changes in immigration patterns could alter our case count predictions. In order to assess the influence of the population base size on TB case count predictions, population denominators from the U.S. Census Bureau’s Current Population Survey (http://www.census.gov/cps/) were incorporated into analysis using 2000–2010 TB case rates. We predicted a decline in TB case rates between 2010 observed and 2020 predicted for all persons and persons who were U.S.-born, foreign-born, foreign-born from the top 5 countries, foreign-born from countries other than the top 5, and foreign-born from Mexico, the Philippines, Vietnam, India and China (data not shown). The predicted declines in TB rates are consistent with our TB case count prediction results.

TB prevention and control strategies, which focus on interrupting recent transmission, have been successful in reducing the number of U.S.-born TB cases but have had limited impact on preventing disease among foreign-born persons entering the United States from countries with high rates of LTBI [21]. LTBI testing and treatment of foreign-born persons from the top 5 countries of birth, particularly those persons who have recently arrived in the U.S., would likely increase the rate of decline of TB cases among foreign-born persons [21]. A newly recommended LTBI treatment regimen [23], which reduces treatment time from 9 months to 12 weeks, increased LTBI treatment completion rates among study populations [24], [25] and could increase LTBI treatment completion in the future as the new regimen is implemented. Unfortunately recent shortages of tuberculin skin test antigens, used for detection of LTBI, and isoniazid, an important drug in the treatment of LTBI and TB disease, have restricted LTBI testing and treatment efforts in some states [26], [27]. Continuation of these shortages could lead to underdiagnoses, incomplete administration of medication and treatment completion failure resulting in an increase in new, recurrent and drug resistant TB disease. Challenges such as these may continue to impact the successful diagnosis and treatment of LTBI and TB disease in the future.

In 2007, the Centers for Disease Control and Prevention issued updated Technical Instructions (TIs) for TB screening of foreign-born persons entering the United States [28]. The updated TIs include guidance on performing TB culture to enhance TB diagnosis and administering directly observed therapy to increase treatment completion. Mexico and the Philippines began screening all applicants to the United States using the updated TIs in 2007, Vietnam in 2008, China in 2009 and India in 2010. A preliminary study of persons immigrating to the United States from Mexico, the Philippines and Vietnam suggests that the updated TIs improved case detection prior to U.S. entry [29]. Evaluation of the newly implemented TIs will be important in determining their impact on TB cases among the foreign-born.

Foreign-born persons who arrive in the United States without going through the immigrant and refugee application process (for example nonimmigrant visitors such as tourists and students, and undocumented foreign-born persons) would not be evaluated using the TIs and thus not be screened for TB regardless of their country of birth. A recent study estimated that between 2001 and 2008 56% of TB cases among newly arrived foreign-born persons were among nonimmigrant visitors who did not undergo TB screening prior to arrival [30]. Furthermore, in 2008 approximately 30% of foreign-born persons in the U.S. were undocumented [31]. Targeted overseas screening of nonimmigrant visitors from countries with a high burden of TB who plan to stay in the U.S. for a long period of time could decrease the number of TB cases among foreign-born persons [30].

The TB prediction methodology described here did not take into consideration factors that affect TB trends among foreign-born persons in the United States such as TB prevalence in the country of birth, immigration patterns, socioeconomic and health status, and global TB control efforts, nor the potential for testing and treatment disruptions due to shortages noted previously. In addition, although the relationship between Human Immunodeficiency Virus (HIV) infection and TB is well described [32], we were not able to incorporate information on HIV status due to underreporting and lack of published HIV incidence and prevalence estimates in the United States by country of origin. We assumed current trends in TB cases will continue with cases declining in a linear pattern; however, trends can be affected by changes in behavior, policies, or other factors that influence population and immigration [20], [22]. Although the linear regression model was not a good fit for all countries, we showed that our prediction results were generally conservative in relation to more complex regression models.

The described prediction analysis had several strengths. The model can be customized for different data subsets, and can be applied to other diseases and conditions. The World Health Organization uses a similar methodology to forecast TB prevalence and mortality rates [10]. Recent data show that our model by country of origin accurately predicted 2011 TB case counts; 2011 observed case counts for all subgroups were within the 95% prediction intervals calculated by our prediction model [12]. Results from our model closely reflect U.S.-born and foreign-born findings from a deterministic mathematical model recently published [4], with the additional benefit that we provide detail by country of origin.

### Conclusions

As a higher proportion of U.S. TB cases occur among foreign-born persons, predicting foreign-born cases will assist TB control programs in using limited resources more efficiently. Our predictions combined with ethnographic information can be used in the development and implementation of effective TB control activities. For example, ethnographic research suggests some Mexico-born populations believe TB can be transmitted through clothing, eating utensils, and body fluids [33], thus education about airborne transmission is a useful component of TB control efforts in this community. CDC has developed ethnographic guides for different foreign-born populations to aid healthcare practitioners in developing culturally appropriate TB care and services (http://www.cdc.gov/tb/publications/guidestoolkits/EthnographicGuides/default.htm).

Our study is one of few that predict future numbers of TB cases in the United States, and the only one to our knowledge that predicts TB case counts among foreign-born persons from specific countries of birth. Further studies that include socioeconomic and demographic factors, TB and HIV burden in countries of birth, and foreign-born immigration trends would enhance TB case count predictions and provide valuable information for TB control programs. Improving TB control among foreign-born persons is imperative as the United States strives to prevent TB transmission and meet elimination goals [34].
